# A Novel Mutation of the* CYP11B2* in a Saudi Infant with Primary Hypoaldosteronism

**DOI:** 10.1155/2017/8431475

**Published:** 2017-10-23

**Authors:** Lama Alfaraidi, Abrar Alfaifi, Rawan Alquaiz, Faten Almijmaj, Horia Mawlawi

**Affiliations:** ^1^College of Medicine, King Saud University, Riyadh, Saudi Arabia; ^2^Department of Pediatrics, Prince Sultan Military Medical City, Riyadh 11159, Saudi Arabia

## Abstract

Isolated hypoaldosteronism is a rare autosomal recessive disease presenting with severe salt wasting and failure to thrive in infancy. A 6-month-old Saudi girl born to consanguineous parents was referred from primary health care for failure to thrive and developmental delay. Laboratory tests revealed hyponatremia, hyperkalemia, and metabolic acidosis with high renin and low aldosterone. Blood samples were collected for endocrine and genetic studies. Sequence analysis of the* CYP11B2* revealed a T to A transition at position 1398 + 2 in exon 8 of the gene in a homozygous state (c.1398+T>A). This result was confirmed by sequencing an independent PCR product. Given the position of the transition at a highly conserved nucleotide and the predictions of different bioinformatic algorithms, it is likely that the mutation is the pathogenic cause of this condition. This result was compared with the reference NM_000498.3. Here, we report a novel homozygous mutation resulting in aldosterone synthase deficiency. To the best of our knowledge, this mutation has not been described in the literature or in any database thus far. The mutation manifested as a rare inherited disease in an infant exhibiting critical salt loss. An adequate replacement treatment will give a good long-term prognosis.

## 1. Background

Aldosterone is a hormone exhibiting potent mineralocorticoid activity that is synthesized in the zona glomerulosa of the adrenal gland and is responsible for maintaining electrolyte balance and intravascular volume [1].

*CYP11B1* (11b-hydroxylaze) and* CYP11B2* (aldosterone synthase), which share more than 90% of their amino acid sequences, encode the enzymes responsible for the three terminal steps in aldosterone formation [2].* CYP11B2* is located on chromosome 8q24 and catalyzes three reactions: the 11-hydroxylation of deoxycorticosterone (DOC) to corticosterone, the 18-hydroxylation of corticosterone to 18-hydroxycorticosterone (18-OHB), and the 18-oxidation of 18-hydroxycorticosterone to aldosterone [3, 4].

Congenital isolated hypoaldosteronism, formerly known as corticosterone methyloxidase deficiency (CMO), is a rare autosomal recessive disorder caused by aldosterone synthase deficiency (ASD) [3]. Most cases have been attributed to approximately 80 different mutations within* CYP11B2*. Patients with this deficiency experience recurrent dehydration, salt wasting, and failure to thrive [4]. ASD is subdivided into CMO types I and II, both of which are characterized by low aldosterone levels and elevated renin activity, accompanied by the accumulation of steroid precursors prior to a biosynthetic block. CMO I is characterized by low levels of 18-hydroxycorticosterone, whereas CMO II is characterized by high levels of 18-OHB and an increase of the urinary excretion of the major metabolite of 18-OHB [3].

ASD has been identified in Jews of European, North American, and Iranian descent [3]. In Asians, it has been reported among Thai [5], Indian [5], and Japanese [6] populations. In this paper, we present a case of isolated primary hypoaldosteronism in a 6-month-old Saudi female infant and the results of her* CYP11B2* analysis.

## 2. Case Report

A female infant born to consanguineous Saudi parents presented at 6 months of age to primary health care workers with failure to thrive and developmental delay. Routine laboratory investigations revealed hyponatremia with hyperkalemia. The infant was delivered prematurely by C-section at 32 weeks with a birth weight 2.4 kg. The child was referred to the Prince Sultan Medical Military Hospital Emergency Center for treatment by an endocrinologist.

On admission, the patient was not dehydrated, and the vital signs were normal. Her weight and length were both below 3rd centile, weight was 3.6 kg (−5.2 SD), and length was 55 cm (−3.9 SD) (Table 1). The systemic examination results were normal; no hyperpigmentation was observed, and the external genitalia were normal. Regarding the patient's development, she was experiencing global developmental delay. She was not able to support her head or hold objects, was not cooing or laughing, had no social smile, and did not recognize her mother. The results of initial investigations were as follows: Na 128 mmol/L, K 7 mmol/L, corrected calcium (CC) 2.6 mmol/L, bicarbonate 15.5 mmol/L, and normal renal function. An adrenocorticotropic hormone (ACTH) stimulation test was performed: before test, the cortisol level was 761 nmol/L and the ACTH level was 5.6 nmol/L (normal), while after test, the cortisol level was 1305 nmol/L, the dehydroepiandrosterone level was 0.118 nmol/L (0.090–3.350), the renin level was 3310 pmol/L (0.15–3.53), and the aldosterone level was 68.4 (139–3660 pmol/L). A 17-hydroxyprogesterone test was normal (Table 2).

Based on the infant's clinical presentation and test results, she was diagnosed with primary hypoaldosteronism. The patient improved clinically, both growth and psychomotor development have matched her chronological age of 2 years, and biochemically when treated with 0.1 mg fludrocortisone twice daily and 1 ml NaCl delivered orally three times per day.

## 3. Methods

### 3.1. Genetic Study

**Figure 1:**
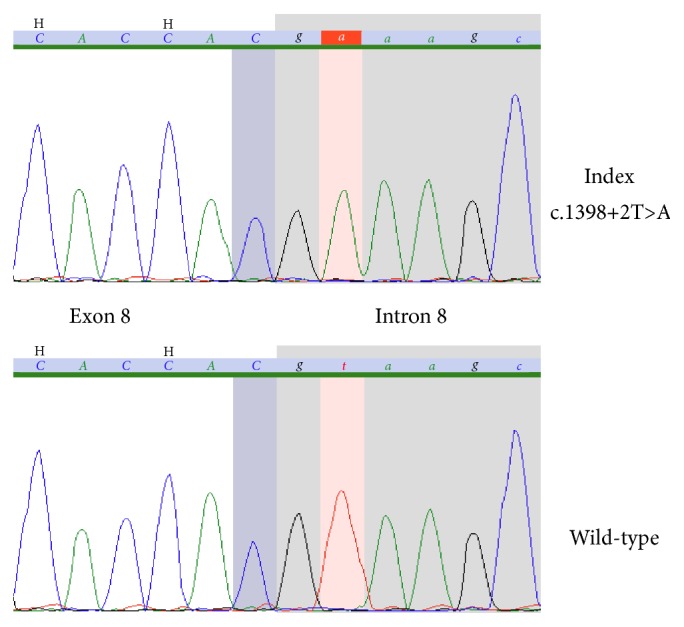
Chromatography.

Molecular genetic analysis of* CYP11B2* was performed. Written informed consent was obtained from the patient's parents. A blood sample was obtained from which DNA was extracted, and molecular genetic analysis of* CYP11B2* was performed.* CYP11B2* (OMIM 124080) exons 1–9 and their respective exon-intron boundaries were amplified by PCR and analyzed by direct sequencing. The resulting sequence data were compared with the reference sequence (NM_000498.3). The patient carried the homozygous mutation c.1398+2T>A(p.?) in* CYP11B2* (Figure 1). The result was confirmed by sequencing of an independent PCR product. Prediction of potential pathogenic effect of the detected mutation was performed utilizing ESE finder (http://krainer01.cshl.edu/cgi-bin/tools/ESE3/esefinder.cgi) [7] and Fruit Fly (http://www.fruitfly.org/) [8].

## 4. Discussion

We describe an infant girl who presented with failure to thrive, poor feeding, and developmental delay. Laboratory tests revealed hyponatremia and hyperkalemia, metabolic acidosis, normal cortisol levels, and high renin and low aldosterone levels. Genetic testing confirmed a diagnosis of primary hypoaldosteronism. The patient improved dramatically after treatment with fludrocortisone.

The ASD* (CYP11B2)* encodes the steroid 11/18 B hydroxylase and is expressed in the zona glomerulosa of the adrenal gland, where it synthesizes the mineralocorticoid aldosterone. ASD type I (CMO type I) is caused by a deficiency in the 18-hydroxylase enzyme, which results in low levels of OHB and aldosterone and low urinary metabolites; in ASD type II (CMO II), 18- OHB levels are markedly elevated, and the levels of aldosterone and its urinary metabolites are low [3]. The plasma renin activity levels are low in both disorders. The ratio of plasma 18- OHB/aldosterone can be differentiated between the two disorders, but this biochemical phenotype has overlapping features and is better considered a continuous spectrum of the same disease [5, 9].

Patients with ASD experience recurrent dehydration, salt wasting, and failure to thrive [4]. The clinical picture varies with age and is most severe during infancy. The severity of salt wasting decreases with age, but the abnormal steroid pattern persists throughout life, possibly due to increases in mineralocorticoid sensitivity and sodium intake with age [10]. However, the initial presentation of life-threating salt wasting that is a hallmark of ASD can be differentiated biochemically from defects in steroid biosynthesis, such as congenital adrenal hyperplasia with salt loss [11].

Primary hypoaldosteronism can be caused by different defects in* CYP11B2*, such as nonsense, missense, and frame shift mutations [10, 11]. However, in this case, we discovered a novel mutation in* CYP11B2* that caused aldosterone deficiency. Genetic analysis revealed a T to A transition at position c.1398+2 in the homozygous state (c.1398+2T>A). Given the position of the transition at a highly evolutionarily conserved nucleotide and the predictions of different bioinformatic algorithms, it is likely that the mutation is pathogenic. The nucleotide exchange is located in intron 8 and affects the donor splice site of exon 8. A computer-based comparison of the modified donor splice site of exon 8 of* CYP11B2* with the wild-type sequence was performed using different bioinformatic tools. This analysis revealed a loss of the constitutive donor splice site of exon 8 due to the alteration c.1398+2T>A. Although sequencing analysis cannot exclude a large heterozygous deletion in the CYP11B2 gene in trans to c.1398+2T>A (i.e., hemizygosity), homozygosity of c.1398+2T>A is most likely.

Both homozygosity and hemizygosity of c.1398+2T>A in the CYP11B2 gene would be compatible with the clinical diagnosis of primary hypoaldosteronism in this patient. To distinguish between homozygosity and compound heterozygosity for c.1398+2T>A with a large deletion comprising this position on the other allele, we are in the process of sequencing the parents' DNA for the mutation.

## 5. Conclusion

To the best of our knowledge, this is the first reported case of a Saudi infant with aldosterone synthase deficiency due to a homozygous alteration (c.1398+2T>A) in* CYP11B2* that has not been described in the literature or any databases thus far. Although it is a rare inherited disease, there is a high index of suspicion of cases with life-threating salt wasting in infancy because of dramatic clinical improvements and good long-term prognosis can be achieved with replacement treatment.

## Figures and Tables

**Table 1:** Growth parameters progression.

Growth	On admission	After 6 months	After 1 year	Recently
parameters	at 6 months	at 12 months	at 18 months	at 2 years
Weight	−5.20 SD (3.6 kg)	−2.80 SD (7.1 kg)	−0.64 SD (10.3 kg)	−0.67 SD (11.3 kg)
Height	−3.90 SD (55 cm)	−1.99 SD (68 cm)	−1.00 SD (77 cm)	−0.40 SD (84.7 cm)

**Table 2:** Laboratory investigations.

Test	Result	Normal reference level
Sodium	128 mmol/L	135–145 mmol/L
Potassium	7 mmol/L	3.6–5.2 mmol/L
Corrected calcium	2.6 mmol/L	2.1–2.6 mmol/L
Bicarbonate	15.5 mEq/L	22–28 mEq/L
Cortisol (prestimulation)	761 nmol/L	140–700 nmol/L
Cortisol (poststimulation)	1305 nmol/L	
ACTH	5.6 Pmol/L	1.6–13.9 Pmol/L
Dehydroepiandrosterone	0.118 nmol/L	0.090–3.350 nmol/L
17-Hydroxyprogesterone	2.3 mmol/L	0–5 nmol/L
Renin	3310 pmol/L	0.15–3.53 pmol/L
Aldosterone	68.4 pmol/L	139–3660 pmol/L

## Additional Points

*Limitations.* We could not differentiate between biochemical types of ASD due to limited resources. The results of genetic testing of the parents are forthcoming.
